# Stromal Cells Derived from Visceral and Obese Adipose Tissue Promote Growth of Ovarian Cancers

**DOI:** 10.1371/journal.pone.0136361

**Published:** 2015-08-28

**Authors:** Yan Zhang, Aleksandra Nowicka, Travis N. Solley, Caimiao Wei, Aaroh Parikh, Laurence Court, Jared K. Burks, Michael Andreeff, Wendy A. Woodward, Ali Dadbin, Mikhail G. Kolonin, Karen H. Lu, Ann H. Klopp

**Affiliations:** 1 Department of Radiation Oncology, The University of Texas MD Anderson Cancer Center, Houston, Texas, United States of America; 2 Department of Gynecologic Oncology, The University of Texas MD Anderson Cancer Center, Houston, Texas, United States of America; 3 Department of Biostatistics, The University of Texas MD Anderson Cancer Center, Houston, Texas, United States of America; 4 Department of Physics and Astronomy, Rice University, Houston, Texas, United States of America; 5 Department of Leukemia, The University of Texas MD Anderson Cancer Center, Houston, Texas, United States of America; 6 The Brown Foundation Institute of Molecular Medicine, The University of Texas Health Science Center at Houston, Houston, Texas, United States of America; Cedars-Sinai Medical Center, UNITED STATES

## Abstract

Obesity, and in particular visceral obesity, has been associated with an increased risk of developing cancers as well as higher rates of mortality following diagnosis. The impact of obesity on adipose-derived stromal cells (ASC), which contribute to the formation of tumor stroma, is unknown. Here we hypothesized that visceral source and diet-induced obesity (DIO) changes the ASC phenotype, contributing to the tumor promoting effects of obesity. We found that ASC isolated from subcutaneous (SC-ASC) and visceral (V-ASC) white adipose tissue(WAT) of lean(Le) and obese(Ob) mice exhibited similar mesenchymal cell surface markers expression, and had comparable effects on ovarian cancer cell proliferation and migration. Obese and visceral derived ASC proliferated slower and exhibited impaired differentiation into adipocytes and osteocytes *in vitro* as compared to ASC derived from subcutaneous WAT of lean mice. Intraperitoneal co-injection of ovarian cancer cells with obese or visceral derived ASC, but not lean SC-ASC, increased growth of intraperitoneal ID8 tumors as compared to controls. Obese and V-ASC increased stromal infiltration of inflammatory cells, including CD3+ T cells and F4/80+ macrophages. Obese and visceral derived ASC, but not lean SC-ASC, increased expression of chemotactic factors IL-6, MIP-2, and MCP-1 when cultured with tumor cells. Overall, these results demonstrate that obese and V-ASC have a unique phenotype, with more limited proliferation and differentiation capacity but enhanced expression of chemotactic factors in response to malignant cells which support infiltration of inflammatory cells and support tumor growth and dissemination.

## Introduction

Obesity increases the risk and/or mortality of many cancers, including endometrial, colon, pancreatic, and ovarian cancers [1–5]. Excess visceral white adipose tissue (WAT) has been shown to be particularity toxic, increasing risk and mortality independent of body mass index (BMI) in many cancers, including ovarian cancer [5]. A number of mechanisms have been identified to account for the relationship between obesity and cancer, such as secretion of adipokines, insulin resistance, and aromatization of steroid hormones to increase circulating estrogen [6–8]. Adipose tissue also contains a population of tumor-tropic adipose stem cells (ASCs) that support the formation of tumor vasculature [9] [10]. Recently, we reported that human omentum-derived ASC promote the growth and vascularization of endometrial cancer xenografts, compared with subcutaneously derived ASC[11]. However, differences in the isolation approach or individual variability may have impacted the ASC phenotype. Furthermore, studies in xenograft models don’t recapitulate the effects of inflammatory cells in the tumor microenvironment, which are better modeled in a syngeneic model. Omentum, part of visceral fat, is the most frequently involved site of ovarian cancer metastasis. Omental metastasis is a particularly critical issue in ovarian cancer, which has the highest recurrence rate and lowest survival among gynecologic cancers. The mechanism behind this is not well known. Besides, syngeneic models of intraperitoneal dissemination are well established in ovarian cancer but not endometrial cancer. Therefore, to understand the role of ASC from different anatomically locations: subcutaneous and visceral adipose tissue, in ovarian cancer, we investigated the effect of diet-induced obesity (DIO) on these different ASCs and their role in tumor growth in the abdominal area by using a syngeneic intra-abdominal murine model of ovarian cancer. *In vitro* and *in vivo* assays were used to analyze ASC isolated from subcutaneous (SC-ASC) and visceral (V-ASC) WAT of lean (Le) and obese (Ob) mice to characterize the effects of obesity and adipose depot and ASC phenotype.

## Materials and Methods

### Cell culture

ASC were grown in α-minimum essential medium (α-MEM) containing 20% FBS, L-glutamine, and penicillin streptomycin. ID8 and IG10 cells were generously provided by Katherine Roby [12]. ID8 and IG10 cells were stably transfected with firefly luciferase and tomato-red genes with use of a lentiviral method[13](pFULT vector was kindly provided by Dr.Jennifer Prescher). Cultured cells were routinely tested for viability with trypan blue exclusion and maintained high viability (>95%). For *in vivo* experiments, the minute fraction of dead cells was predominantly removed by washing before trypsinization.

### Isolation of SC-ASC and V-ASC

ASC were isolated from subcutaneous WAT and visceral WAT of C57BL/6 female mice fed with low-fat diet (LFD) or high-fat diet (HFD) for 15 weeks. HFD and LFD were purchased from Research Diet INC. Subcutaneous WAT was taken from the posterior torso, and visceral WAT was taken from retroperitoneal and gonadal depots. WAT was subjected to mechanical disruption, followed by 0.5 mg/mL collagenase type I (Worthington Biochemical) and 50 U/mL dispase (Becton Dickinson) digestion according to published protocols [11]. Digested WAT was centrifuged at 1,000 rpm for 5 minutes. Supernatant containing adipocytes was removed. The resulting cell pellet was resuspended in α-MEM containing 20% FBS and filtered through 100 μm cell strainer (Becton Dickinson) and then through 40-μm cell strainers.

### Characterization of SC-ASC and V-ASC

All ASC were expanded *in vitro* and early passaged ones were used to characterize with use of flow cytometry for expression of the following cell surface markers: CD34, CD31, CD45, CD29, CD11b, CD73, CD90, and CD105 (Becton Dickinson). Cell differentiation studies were done as described previously [14]. For adipocyte differentiation, confluent cells in 6-well or 24-well plate were cultured in adipogenic induction medium, Dulbecco's Modified Eagle's Medium (DMEM; Mediatech) supplemented with 10% FBS, 1% penicillin/streptomycin, 10 μg/mL insulin, 500 μM 3-isobutyl-1-methylxanthine (Sigma-Aldrich), 1 μM dexamethasone (Sigma-Aldrich), and 200 μM indomethacin (Sigma-Aldrich). After cells were maintained in induction medium for 72 hours, the medium was changed to adipogenic maintenance medium, Dulbecco's Modified Eagle's Medium supplemented with 10% FBS, 1% penicillin/streptomycin, and 10 μg/mL insulin for 24 hours. This procedure of 72 hours induction and 24 hours maintenance was repeated twice. After the third induction cells were placed in maintenance medium for a total of 10 days of incubation. Maintenance media was changed twice a week. Cells were then fixed in 10% formalin for 10min followed by 60% isopropanol. Oil Red O (Sigma-Aldrich) staining was applied to visualize red lipid vacuoles. For osteoblast differentiation, confluent cells in 6-well plates were cultured in NH OsteoDiff Medium (Miltenyi) for 3 weeks, with the medium changed twice a week. After 3 weeks, cells were washed 3 times with PBS and fixed with precooled 100% methanol. Cells were stained with Alizarin Red S. Five representative Images from each well (three wells for each ASC) were taken by Leica DMI6000B microscope and analyzed by Inform software: regions of red Alizarin or Oil Red O stained were defined on 4–6 images, and the recognition software was applied to all images. The staining in pixels was quantified for each image, and the percentage of staining was calculated and averaged from all images for a final percentage.

### qPCR assay

mRNA from the Ob/Le derived SC-ASC or V-ASC at different days during adipogenesis induction was isolated using TRIzol and converted to cDNA using SuperScript III Reverse Transcriptase (Invitrogen). Quantitative RT-PCR analysis was performed based on TaqMan primers/Universal PCR Master Mix (Invitrogen)platform using ABI 7900 workstation and SDS 2.4 software (Applied BioSystems, Grand Island, NY, USA) and 2^-delta Ct value was used for the analysis[15].

### Proliferation assay

Luciferase-labeled ID8 and IG10 cells were plated at a density of 500 cells per well in a BD Falcon 96-well plate alone or in 1:1 coculture with early passaged ASC. After 24 hours, luciferase expression was measured in 0.15 mg/mL D-luciferin. After 1 hour of incubation at 37°C, luminescence was measured with a spectrophotometer (FLUOstar Omega, BMG Labtech). The medium was then replaced, and measurements were repeated every other day until cells reached confluence.

### Migration assay

Conditioned Medium (CM) from early passaged subcutaneous or visceral ASC of obese or lean WAT was collected from a 6-well plate that was plated with 300,000 cells per well with serum-free medium for 48 hours. ID8 and IG10 migration was analyzed in ASC CM in a 24-well transwell (Corning Inc.) with 8-μm pores. CM was placed in the lower portion of the transwell and ID8 on the upper portion of the transwell. Each CM was assayed in triplicate. The number of cells that had migrated to the bottom of the transwell was measured after 8 hours by staining membranes with a Hema 3 Stat Pack (Thermo Fisher Scientific) and then mechanically removing residual cells on the upper membrane. Cells attached to the bottom of the membrane were dissociated from the membrane using 2% deoxycholicacid. The optical density of the resulting suspension was detected by an enzyme-linked immunosorbent assay (ELISA) plate reader (BioTek Instruments) at 590nm in a 96-well plate [16].

### Spheroid formation assay

A total of 40,000 early passaged ASC or ovarian cancer cells were plated in 2 mL of spheroid assay medium (MEM with 0.1 mg/mL gentamycin, 1% penicillin/streptomycin, 10 mL of B27, 10 μg of FGF, and 10 μg of EGF from Gibco) in low attachment plates for 5 days and collected as spheriod-conditioned medium (spheroid-CM). Spheroid cultures were initiated by seeding 100 cancer or ASC cells in 100ul of medium consisting of 50% ASC or cancer cell spheroid-CM and 50% regular spheroid assay medium in 96-well plates. Fourteen days later, 7.5 μL of MTT (Methylthiazolyldiphenyl-tetrazolium bromide) was added to each well. Spheres were counted manually with diameter of at least 50um to exclude single cells or debris. Each sample was repeated in 8 wells.

### Luminex assays

The spheroid CM described above was analyzed for angiogenesis/growth or cytokine/chemokine factor panel including IL-6, SDF-1, MCP-1, TNF-a, IL-10, MIP-1a, 1b, MIP-2, and VEGF with Luminex 100 by using a Milliplex MAP Kit (EMD Millipore Corp, Billerica, MA, USA). Data were processed by Bioplex manager software.

### *In vivo* studies

Animal experiments were conducted according to MD Anderson Institutional Animal Care and Use Committee–approved protocol (120914932). All mice were housed and treated in accordance with institutional standards. Mouse strains C57BL/6 were from Jackson Lab. For DIO induction [17], HFD D12492 (60 kcal% fat) and LFD D12450B (10 kcal% fat) from Research Diets were used. All the mice in our experiments were given 2–4% isoflurane for anesthetizing and the depth of anesthesia was monitored by respiratory rate and absence of withdrawal reflex to footpad pinch. To evaluate the effect of obesity on cancer a total of 10^6^ luciferase-expressing ID8 in 200 μL of PBS were injected intraperitoneally (IP) into HFD or LFD mice, and tumors were grown for 10 weeks obesity for tumor growth. In order to investigate further the different affects of visceral derived ASC and subcutaneous derived ASC on cancer, 6-week-old lean mice were anesthetized as before and injected intraperitoneally with 1 × 10^6^ luciferase-expressing ID8 cells and 1 x 10^6^ early passages of SC-ASC or V-ASC from obese or lean mice into the abdomen (5 mice per group). Luciferase signals were measured to determine the tumor size by IVIS Imaging system 200 series (Xenogen Corporation) twice a week after 4 days, and images were analyzed using Living Image software (Caliper LifeSciences). At around day 50, mice were euthanized by inhalation overdose of isoflurane, ascites were collected, and tumor nodules near the stomach were recovered from euthanized mice. Ascites were centrifuged, and red blood cell lysis (eBioscience) was used to remove red blood cells in the cell pellets; this was followed by 10 mL of a-MEM with 20% of FBS for neutralization. Cells from ascites were stored at -80°C and were later analyzed by flow cytometry for cell composition. Immunofluorescence on formalin-fixed, paraffin-embedded tissue sections was carried out after antigen retrieval, washing with 0.2% Triton X-100, and blocking in Serum-Free Protein Block (DAKO); this was followed by incubation with primary antibodies (4°C, 12 hours) and secondary antibodies (room temperature, 1 hour) in PBS containing 0.05% Tween-20. The primary antibodies used were as follows: rabbit anti-Ki-67 (Thermo Scientific, 1:200), Biotinylated GSL I–isolectin B_4_ for tumor vessels staining (Vector, 1:50), rat anti-F4/80 (Abcam, 1:100), rat anti-CD3 (Abcam, 1:100), and rabbit anti-perilipin (Cell Signaling Technology, 1:100). Secondary donkey Alexa 488–conjugated (1:150) IgG was from Invitrogen, Cy3-conjugated (1:300) IgG was from Jackson ImmunoResearch, and streptavidin-Cy3 (1:20) was from Invitrogen. Nuclei were stained with Hoechst 33258 (Invitrogen).

### MicroCT Scan on mice SC-WAT and V-WAT

To determine the volume of visceral and subcutaneous adipose tissues, we used MATLAB(Mathworks, Natick, MA) to create an in-house program. MicroCT scans were acquired using (scanner) and taken at (kVp, mA, FOV, slice thickness). CT images for each mouse were imported as a series of DICOM images. The region of interest (ROI) for adipose volume determination was limited to the abdomen. Accordingly, the upper and lower boundaries of the ROI were defined from the bottom of the diaphragm to the superior aspect of the femoral heads. Next, we manually drew elliptical contours inside the abdominal musculature extending posteriorly to the vertebral body so that visceral adipose tissue was contained within the ellipse and subcutaneous adipose tissue was outside the ellipse. The ellipses were drawn on the top, upper quartile, middle, lower quartile, and bottom slices of the ROI. During this process, we determined the optimal location and size of the ellipse in order to separate visceral adipose tissue and subcutaneous adipose tissues as much as possible. The program then linearly interpolated these contours for the remainder of the slices, effectively separating the ROI into 2 regions. The area enclosed by the ellipse contained the abdominal cavity, which included visceral adipose tissue and organs. The area outside of the ellipse contained subcutaneous adipose tissue.

Finally, the program calculated adipose tissue volume by automatically counting the number of voxels corresponding to adipose tissue, and multiplying by the volume of each voxel. Subcutaneous adipose consisted of tissues between – 50 and 150 Houncefield unit (HU) and visceral adipose consisted of tissues between –50 and 200 HU. The ranges were selected based on visual analysis of the HU distribution of adipose tissues.

### Image acquisition and data analysis

Images were acquired by Vectra Automated Multispectral Imaging System (Perkin Elmer) and analyzed by Inform software: regions of interest were defined on 4–6 images, and the recognition software was used to classify all images. Fluorescence of interest in pixels was quantified for the various tumor categories of each image, and the percentage of fluorescence from the target tumor category was calculated and averaged from all images of one tumor for a final percentage. At least 10 images per slide were quantified. Three tumors were analyzed from each of ID8 experimental groups. Statistical analysis was conducted with the Mann-Whitney test, two-tailed.

### Flow cytometry

ASC or cells from tumor or ascites were resuspended in PBS supplemented with 2% FBS (10^6^ cells/100 μL/staining reaction). 1 μg of each antibody was added to the cell suspension and incubated at 4°C for 30 minutes. Labeled cell populations were then analyzed by Gallios flow cytometer (Beckman Coulter) LSRII (BD Bioscience) with Kaluza or FLowJo software. Sample acquisition was accompanied with use of control unstained, single-color stained, and isotype controls to determine the appropriate voltages, compensations, and positioning of gates for data acquisition. Ascites cells were pre-gated to exclude debris, cell clumps, contaminating polymorphonuclear cells, red blood cells, and dead cells based on DAPI staining. Cell compositions were analyzed based on Texas Red (Texas Red channel) and the following IgG clones: APC-anti-CD34 (RAM34), PE-Cy7-anti-CD31 (MEC 13.3), APC-Cy7-CD45 (30-F11), or Texas Red, PE-Cy7-anti-CD11B, and APC-F4/80, and the corresponding isotype controls (BD Biosciences). Isotypes and the positions of previously characterized hematopoietic and endothelial populations on the plots [18,19] were used to set gate cutoffs.

### Statistical analyses

For analysis of *in vivo* data, tumor size measured as luminescence intensity was transformed by base 10 logarithms to approximate normality for linear model. A linear mixed effect model with repeated measures was used to evaluate the differences among groups (Le-V-ASC, Le-SC-ASC, Ob-V-ASC, Ob-SC-ASC, and ID8 only) across multiple time points. Our final model included the fixed effect of group (5 groups), and day (the interaction between group and day was not significant), random effect of mice nested within group, and repeated measures with the first-order auto regressive covariance structure. We examined the normality of the residuals by normal quantile-quantile plot (Q-Q plot) [20]. The analysis of the *in vitro* data was performed using SAS 9.3 (Gary, NC). In vitro results were reported as means +/− standard error of mean. *P* values were obtained by using the two-tailed Mann-Whitney test. The log-rank test was used to compare time to detection of disease by using luciferase imaging.

## Results

### Obesity increases growth of ID8 ovarian cancers

**Fig 1:**
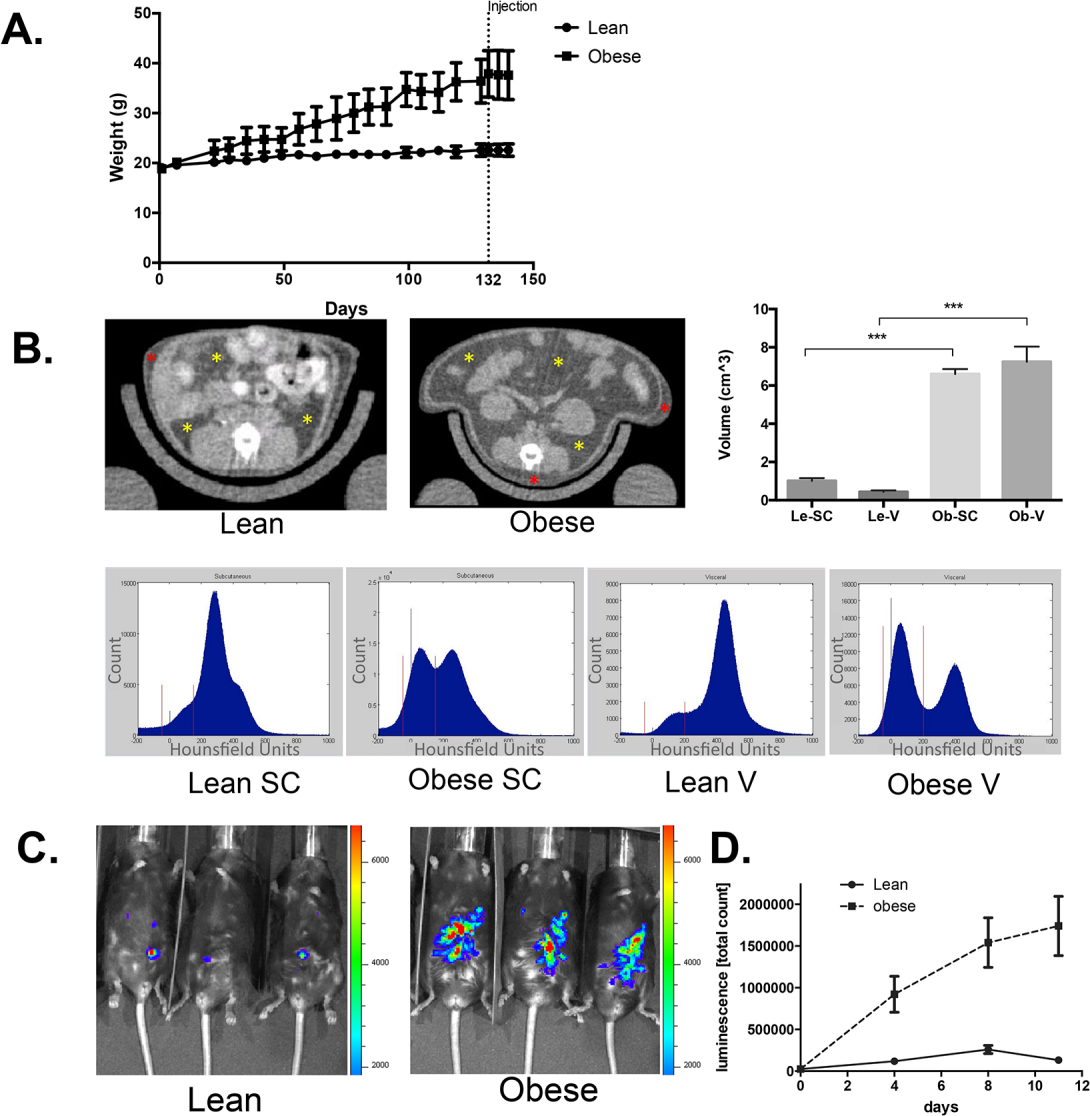
Obesity promotes ID8 tumor growth. **A**, C57BL/6 mice were fed a HFD or LFD for 4 months. N = 10 per group. Mice that were fed a HFD had higher body weight than mice that were fed a LFD (mean body weight, 37.6 g vs. 22.6 g). **B**, CT scans of HFD or LFD mice (HFD: mean visceral WAT: 7.2 cm^3^ and mean SC WAT 6.6 cm^3^; LFD: mean visceral WAT: 0.4 cm^3^ and mean SC WAT 1.0 cm^3^) and HU distribution of visceral and subcutaneous fat from HFD or LFD mice. Yellow asterisks indicate visceral fat, and red asterisk indicates subcutaneous fat. Red vertical lines denote the upper and lower vales that define the HU interval corresponding to adipose tissue. Error bars, SEM. ***, *P* < 0.001(Student *t* test). **C**, Representative HFD and LFD mice with luciferase signals from tumors. Mice were i.p. isografted with 1 x 10^6^ luciferase-expressing ID8 tumor cells. Tumors were measured by luciferase signals detection within abdominal side of mice. **D**, Diet induced obesity accelerated ID8 ovarian tumors. Tumor growth in HFD and LFD mice for 11 days.

To determine whether obesity has a direct effect on the growth of murine ovarian cancer, we used a syngeneic murine model of ovarian cancer in C57Bl/6 mice that are prone to DIO. C57BL/6 female Mice were fed a high-fat diet (60% of kcal from fat) or a low-fat diet (20% of kcal from fat). After 4 months, mice fed with a high-fat diet had nearly twice the body weight of mice fed the low-fat diet (mean body weight, 37.6 g vs. 22.6 g) (Fig 1A). To determine whether mice with DIO had increased visceral WAT as well as subcutaneous WAT, the volume of visceral WAT was measured with microCT scans. Mice with DIO contained more than a 16.7-fold higher volume of visceral fat and 6.5-fold higher volume of subcutaneous fat than lean mice (Fig 1B). Mice were then isografted with 1 x 10^6^ firefly luciferase-expressing ID8 ovarian cancer cells intraperitoneally. At 11 days, luciferase activity was significantly higher in mice with DIO than in lean mice (Fig 1C and 1D), demonstrating that diet-induced obesity accelerates ID8 ovarian tumors.

### Isolation and characterization of ASC from subcutaneous and visceral adipose from lean and obese mice

**Fig 2:**
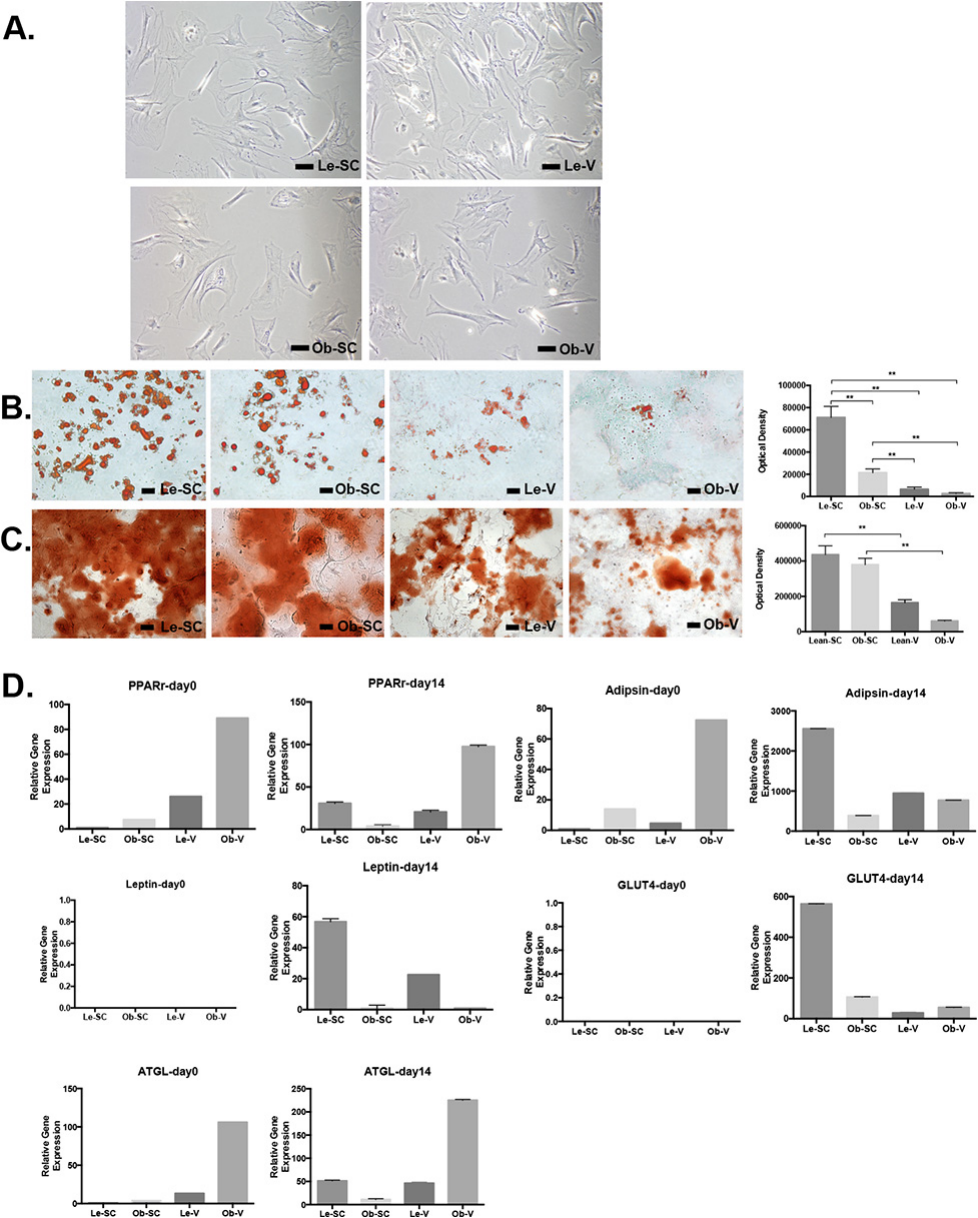
Characterization of SC-ASC and V-ASC from obese and lean mice. **A**, Morphology of adherent cells showing similarity of SC-ASC and V-ASC from obese and lean mice. Scale bar, 1μm. **B**, Adipogenesis of SC-ASC and V-ASC. Differentiated cells were stained with oil red-O and quantified by percentage of pixels of oil red-O in images. Error bars, SEM. **, *P* < 0.01(Mann-Whitney test). Scale bar, 100μm.**C**, Osteogenesis of SC-ASC and V-ASC. Differentiated cells were stained with alizarin red S. Error bars, SEM. **, *P* < 0.01(Mann-Whitney test). Scale bar, 100μm. **D**, Quantitative RT-PCR analysis of mRNA levels for genes hallmarking adipocyte differentiation and lypolysis normalized to 18s RNA expression in indicated ASC at baseline and 14 days after adipogenesis induction. Error bar: SEM.

To determine whether obesity effects were mediated by ASC, we isolated and characterized SC-ASC and V-ASC of tumor-free mice fed a HFD or LFD, as described above. ASC were isolated according to previously published protocols [11]. Morphologically, SC-ASC and V-ASC from obese or lean mice exhibited a similar mesenchymal phenotype (Fig 2A). Cell surface marker expression was characterized in triplicate with flow cytometry after cells were passaged 3 times (S1 Fig and S1 Table). All ASC were negative for endothelial marker CD31, monocyte marker CD11b, and hematopoietic lineage marker CD45. Mesenchymal marker, CD29 was expressed on all 4 ASC lines, while mesenchymal markers CD90 and 105 were present on the majority of SC-ASC and Le-V-ASC but less frequent in Ob-V-ASC. These results demonstrate that there was no evidence of contamination with non-mesenchymal lineage cells from adipose tissues and that SC and Le-V-ASC had similar cell surface marker expression. Ob-V-ASC were noted to have less expression of CD90 and 105 which are characteristic of MSC. Next, to determine whether SC-ASC and V-ASC from obese or lean mice maintain the same growth rate, cell proliferation assays were performed. Lean SC-ASC (Le-SC-ASC) proliferated more rapidly than ASC from other sources (*P* > 0.05, S2 Fig).

### *In vitro* ASC differentiation

To determine whether SC-ASC and V-ASC from obese or lean mice exhibit multipotency *in vitro*, adipocyte and osteocyte lineage differentiation assays were performed as reported previously (Fig 2B and 2C) [11]. Adipocyte quantification was based on Oil Red O staining of lipid droplet after adipogenesis induction. SC-ASC formed more Oil Red O–positive lipid droplets than V-ASC. (Fig 2B, *P* < 0.01). Osteogenesis was detected with use of Alizarin red staining to detect alkaline phosphatase, indicative of osteoblastic differentiation (Fig 2C). Osteogenesis was also more robust for SC-ASC than for V-ASC (*P* < 0.01) with a trend toward obesity impairing osteogenic differentiation potential. Combined, these findings suggest that SC-ASC have higher multipotency than V-ASC and that obese WAT-derived ASC have more limited differentiation potential than lean WAT-derived ASC.

In order to determine if tissue source and obesity impacted adipogenic programming, expression of adipocyte (adipsin, leptin, GLUT4), lipolysis (ATGL) and key adipogenesis regulating transcription factor (PPAR-γ) were profiled in ASC at baseline and following 14 days of adipocyte induction (Fig 2D). We found that Le-SC-ASC underwent 30-fold induction of the critical adipogensis-regulating gene, PPAR-γ following adipocyte induction, unlike ASC from other sources. The level of PPAR- γ was still high after differentiation in Ob-V-ASC group but contained few Oil-Red-O staining (Fig 2C). This may be due to increased lipolysis, which is reflected in higher levels of ATGL expression in Ob-V-ASC. Adipocyte genes including adipsin, leptin and GLUT4 were expressed at much higher levels in Le-SC-ASC, consistent with our observation that SC-ASC differentiated most robustly into adipocytes. Lipolysis regulating gene ATGL was highest in obese visceral derived ASC.

### *In vitro* analysis of ASC and ovarian cancer co-cultures

To determine whether ASC have a direct mitogenic effect on malignant cells, we analyzed proliferation of ID8 and IG10 C57 derived ovarian cancer cell lines *ex vivo*. Luciferase-labeled ID8 and IG10 cells were co-cultured with or without ASC. Tumor cell proliferation was monitored with use of luciferase imaging. ASC from all sources had a modest pro-proliferative effect on ID8 and IG10 cells (S3A and S3B Fig). To determine whether ASC affect the invasive potential of ovarian cancer cells, an *ex vivo* migration through Boyden Chamber Transwell assay was performed. ASC from all sources, compared with ovarian cells alone, increased the migration of ovarian cells (*P* <0.01) (S3C and S3D Fig).

**Fig 3:**
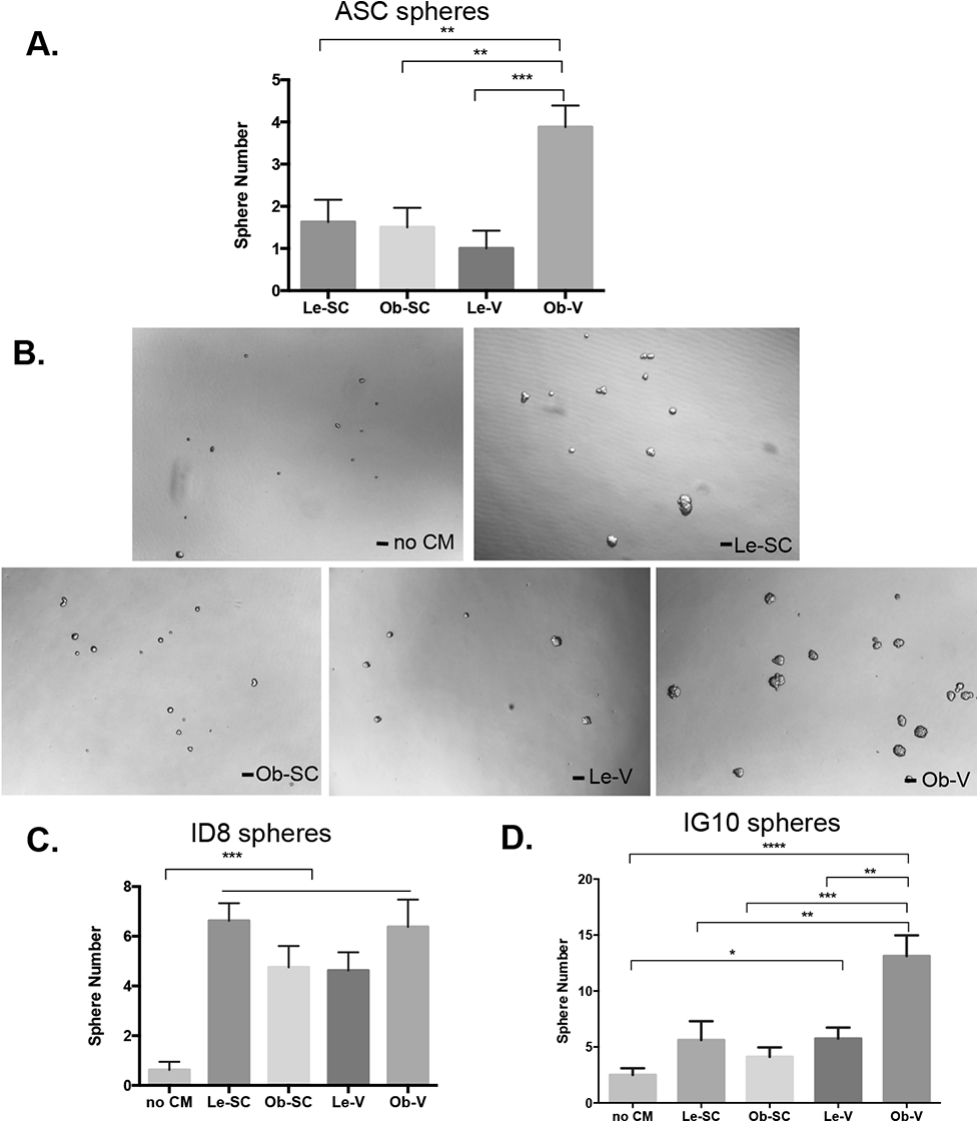
Spheroid formation assay. Cells were plated at a density of 100 cells in total 100ul in 96-well plates and cultured for 14 days. Cells were then stained with MTT, and spheroids were counted manually. Each experiment was performed in triplicate. **A**, Quantification of ASC spheroid in regular medium. **B**, Comparison of IG10 spheroid formation in various ASC spheroid-CM. Representative images shown at 10x magnification. Scale bar, 100μm. **C**, Quantification of ID8 spheroid assay in different ASC spheroid-CM. (Shown are mean ± SEM, ***, *P* < 0.001, Student *t* test, two tailed.). **D**, Quantification of IG10 spheroid assay in various ASC spheroid-CM. (Shown are mean ± SEM, *, *P* < 0.05; **, *P* < 0.01, ***, *P* < 0.001, Student *t* test, two tailed).

Malignant ovarian cancer cells aggregate and form spheroid-like structures in ascites fluid which can facilitate omental metastasis [21]. We have previously reported that bone marrow derived MSC enhance the formation of breast cancer spheroids [22]. When ASC were cultured as spheroids at 100 cells plated per well without tumor cells, Ob-V-ASC formed significantly more spheres that ASC from all other sources (Fig 3A). To determine the effect of ASC on ovarian cancer spheroid formation, ID8 and IG10 cells were grown as spheroids with or without ASC spheroid-CM. ID8 cells formed significantly more spheroids in all ASC-spheroid-CM than ID8 cells alone (Fig 3C, *P* < 0.05). IG10 cells formed significantly more spheroids when cultured with Ob-V-ASC spheroid-CM (Fig 3B and 3D). In summary, obesity and tissue source appear to affect ASC spheroid formation, and support of IG10 ovarian cancer cell derived spheroids.

### Effect of obese and lean WAT derived ASC on *in vivo* growth of ovarian cancer

**Fig 4:**
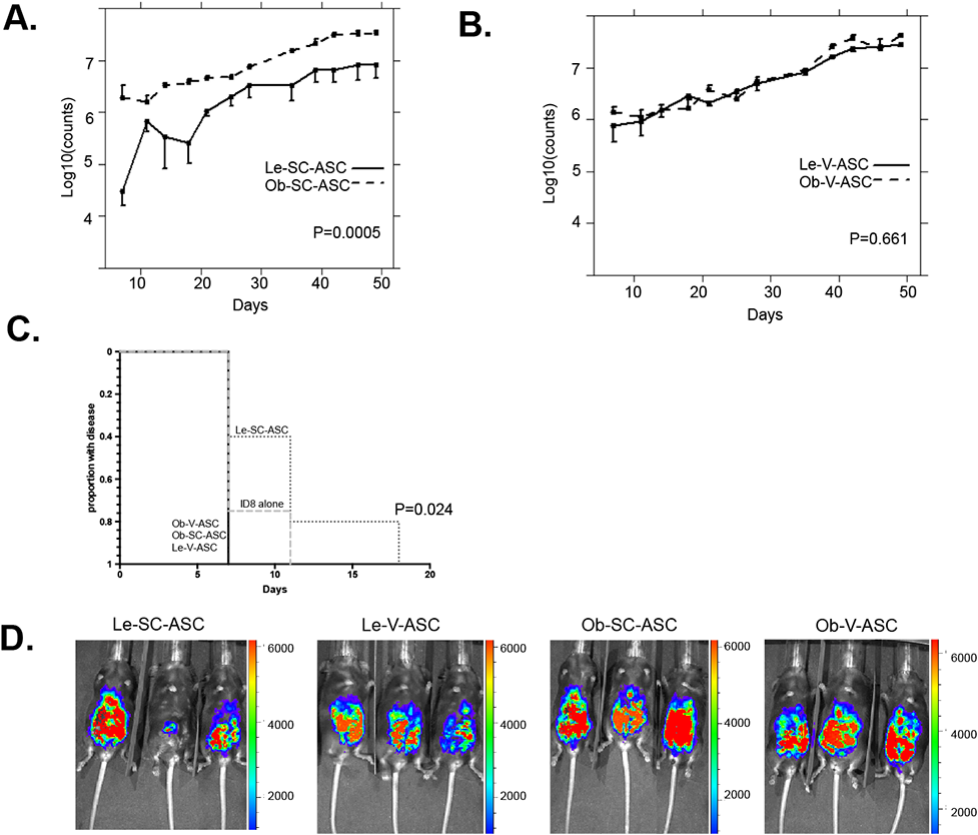
Ovarian tumor growth promotion by ASC. ASC were coinjected with luciferase-expressing ID8 tumor cells into C57BL/6 mice intraperitoneally (1:1 ratio, 10^6^/each). N = 5 per group. **A**, Comparison of tumor burden measured by luciferase expression at abdominal side between Ob-SC-ASC and Le-SC-ASC groups. Error bar: 90% confidence interval. **B**, Comparison of tumor burden measured by luciferase expression at abdominal side between Ob-V-ASC and Le-V-ASC groups. Error bar: 90% confidence interval. **C**, Comparison of tumor initiation among groups. **D**, Luciferase signal measurement of abdominal side in the mice at day 46.

To investigate the *in vivo* effects of tissue source and obesity on ASC in ovarian cancer, ASC were co-injected with luciferase-expressing ID8 tumor cells into lean female C57BL/6 mice intraperitoneally (1:1 ratio, 10^6^/each). Tumor growth was monitored with luciferase imaging. Higher levels of luciferase activity were detected in mice injected with Ob-SC-ASC, Le-V-ASC and Ob-V-ASV as compared to mice injected with ID8 cells alone (Table 1 and Fig 4A–4D). Tumor growth in mice injected with Le-SC-ASC was similar to tumor growth in mice receiving ID8 cells alone. Ob-SC-ASC promoted tumor growth more than did Le-SC-ASC (*P* = 0.049). Ob-SC-ASC and Ob-V-ASC had similar tumor promoting effects (*P* = 0.84). In addition, tumors in mice injected with Le-SC-ASC were detected later than were tumors in mice injected with ASC from other sources (*P* = 0.024, Fig 4C).

**Table 1:** ID8 tumor growth pairwise comparison between groups

Group 1	Group 2	Least squares mean differences between two groups	SE of the difference	P value
ID8 only	Le-SC-ASC	-0.4389	0.3438	0.21
ID8 only	Ob-SC-ASC	-1.1611	0.3433	0.003
ID8 only	Le-V-ASC	-0.9703	0.3433	0.011
ID8 only	Ob-V-ASC	-1.0450	0.3643	0.01
Le-SC-ASC	Ob-SC-ASC	-0.7222	0.3440	0.049
Le-V-ASC	Ob-V-ASC	-0.07462	0.3644	0.84

Shown as Statistic results of ID8 tumor growth between groups based on a linear mixed effect model with repeat measures to evaluate the effect of ASC source (V/SC) and obesity status (obese/lean) versus control group across multiple time points. Note that the difference was between two groups averaged over all time points because there was not significant interaction between treatment group and time point. SE: standard error.

### Effect of ASC on the tumor microenvironment

**Fig 5:**
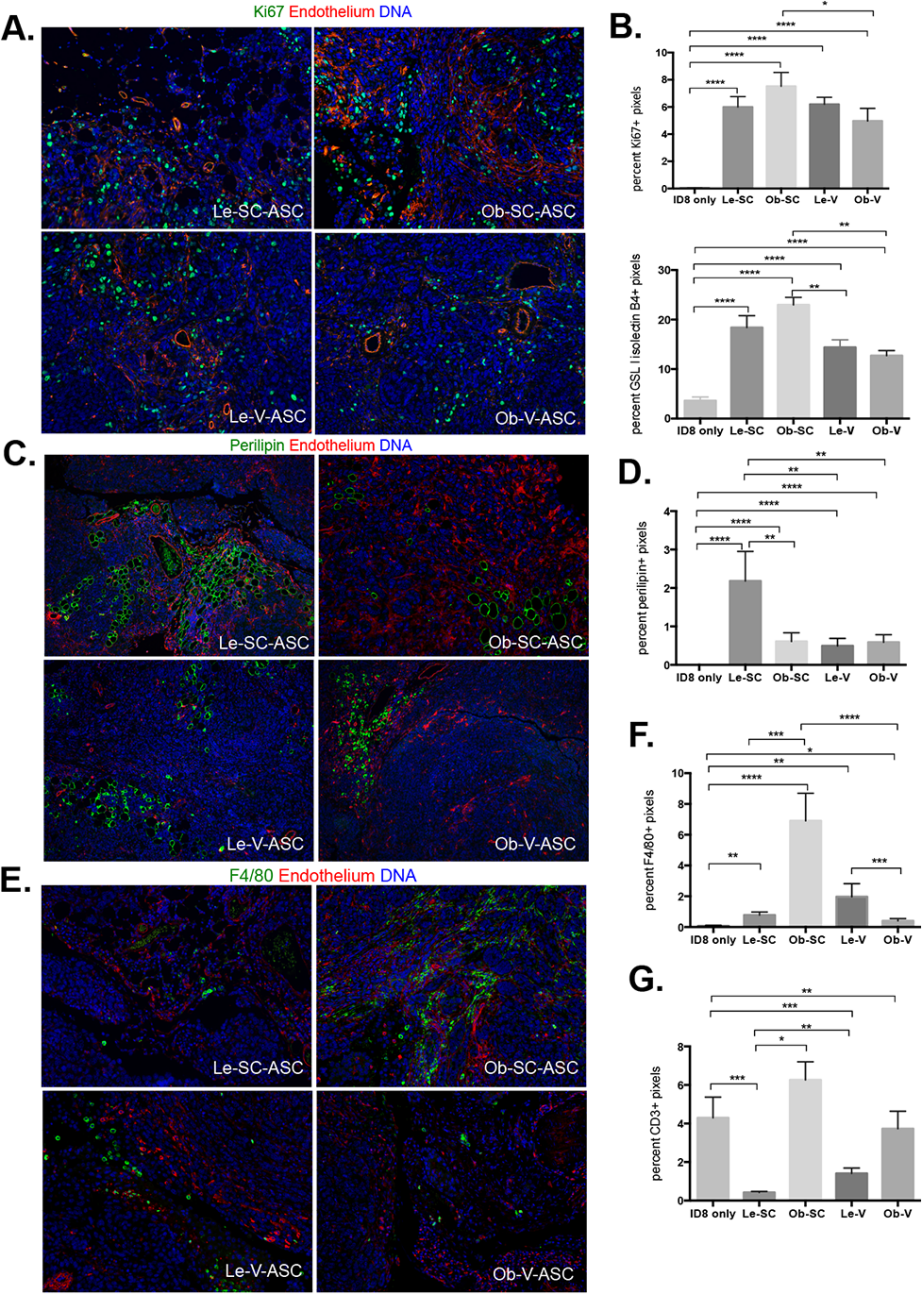
Tumor cell proliferation, vascularization, adipogenesis promotion and immune cells infiltration promotion by ASC. Immunofluorescence analysis of tumors collected at day 49 after mice were euthanized. Nuclei are blue in all images(magnification is 20x) and quantification were done from more than 20 randomly selected images taken by Vectra and analyzed by Inform. **A**, Proliferating cells identified with anti-Ki67 (green) antibody. Vessels were stained with GSL I-isolectin B4 in red. **B**, Quantification of proliferating cells and vessels. Shown are mean ± SEM. *, *P* < 0.05; **, *P* < 0.01,Mann-Whitney test, two tailed. **C**, Adipocytes were stained with anti-perilipin (green) antibody and vessels were stained with GSL I-isolectin B4 in red. **D**, Quantification of adipocyte in the tumors. (Shown are mean ± SEM. A-D, **, *P* < 0.01, Mann-Whitney test, two tailed) **E**, Macrophages stained with anti-F4/80+ (green) antibody varied in the four groups. Vessels were stained with GSL I-isolectin B4 in red. **F**, Quantification of F4/80+ cells in the tumors. (Shown are mean ± SEM. *, *P* < 0.05; ***, *P* < 0.001; ****, P<0.0001. Mann-Whitney test, two tailed) **G**, Quantification of macrophages in the tumors. (Shown are mean ± SEM. *, *P* < 0.05; **, *P* < 0.01; ***, *P* < 0.001; ****, P<0.0001. Mann-Whitney test, two tailed)

To determine the effect of ASC on in-vivo tumor cell proliferation, we quantified the number of Ki-67–positive cells within ID8 tumors with use of immunofluorescence (Figs 5A and S4). The number of pixels positive representing frequency of cells for Ki-67 was higher in ASC injected tumors but there were no different between obese and lean groups (Fig 5A and 5B). Tumors from Ob-SC-ASC group contained slightly higher Ki67 signals than Ob-V-ASC group (P<0.05) (Fig 5A and 5B). We had previously demonstrated that ASC support the formation of vasculature in endometrial xenografts [11]. To determine whether these ASC effects on tumor vascularity are modified by obesity and tissue source, tumor sections were stained with GSL I-isolectin B4, which specifically binds to endothelial cells (Fig 5A). Vascularity was higher in all ASC injected tumors as compared to ID8 tumors alone with a significantly higher number of vessels upon co-injection of Ob-SC-ASC than Le-V-ASC or Ob-V-ASC (*P* < 0.01, Fig 5B).

To determine whether the number of intra-tumoral adipocytes was altered by injection of ASC from lean and obese mice, tumor sections were stained to detect perilipin, a marker for mature lipid droplets (Figs 5C and S4). Quantification assays showed that the Le-SC-ASC group contained significantly more adipocytes than did any of the other three groups (*P* < 0.01, Fig 5D).

To evaluate whether ASC from various sources altered the recruitment of immune cells such as T cells and macrophages, tumor sections were stained with F4/80 (Figs 5E and S4) and CD3 (images not shown), which identify macrophages and T-cells, respectively. ASC from all sources increased macrophage infiltration with Ob-SC-ASC tumors containing significantly more macrophages than the Le-SC-ASC and V-ASC injected mice (*P* < 0.0001, Fig 5F). The number of infiltrating T cells was higher in tumors from mice receiving Ob-SC-ASC than in tumors from mice receiving Le-SC-ASC (*P* < 0.05, Fig 5G). There is a trend that tumors from Ob-V-ASC group contained more T cells than Le-V-ASC. Tumors derived from injection of ID8 cells alone contained a comparable number of T-cells as tumors injected with Ob-SC-ASC and more than lean derived ASC from either source (P<0.001).

In summary, we found that tissue source and obesity alter the impact of ASC within the tumor microenvironment, with a trend of obese-derived ASC increasing the infiltration of T-lymphocytes into ID8 tumors compared with Le-derived ASC. In general, ID8 tumors from mice receiving Le-SC-ASC were less likely to contain inflammatory cells and more likely to be populated with intratumoral adipocytes.

### Induction of chemotactic cytokine expression in ASC co-cultured with ovarian cancer cells

**Fig 6:**
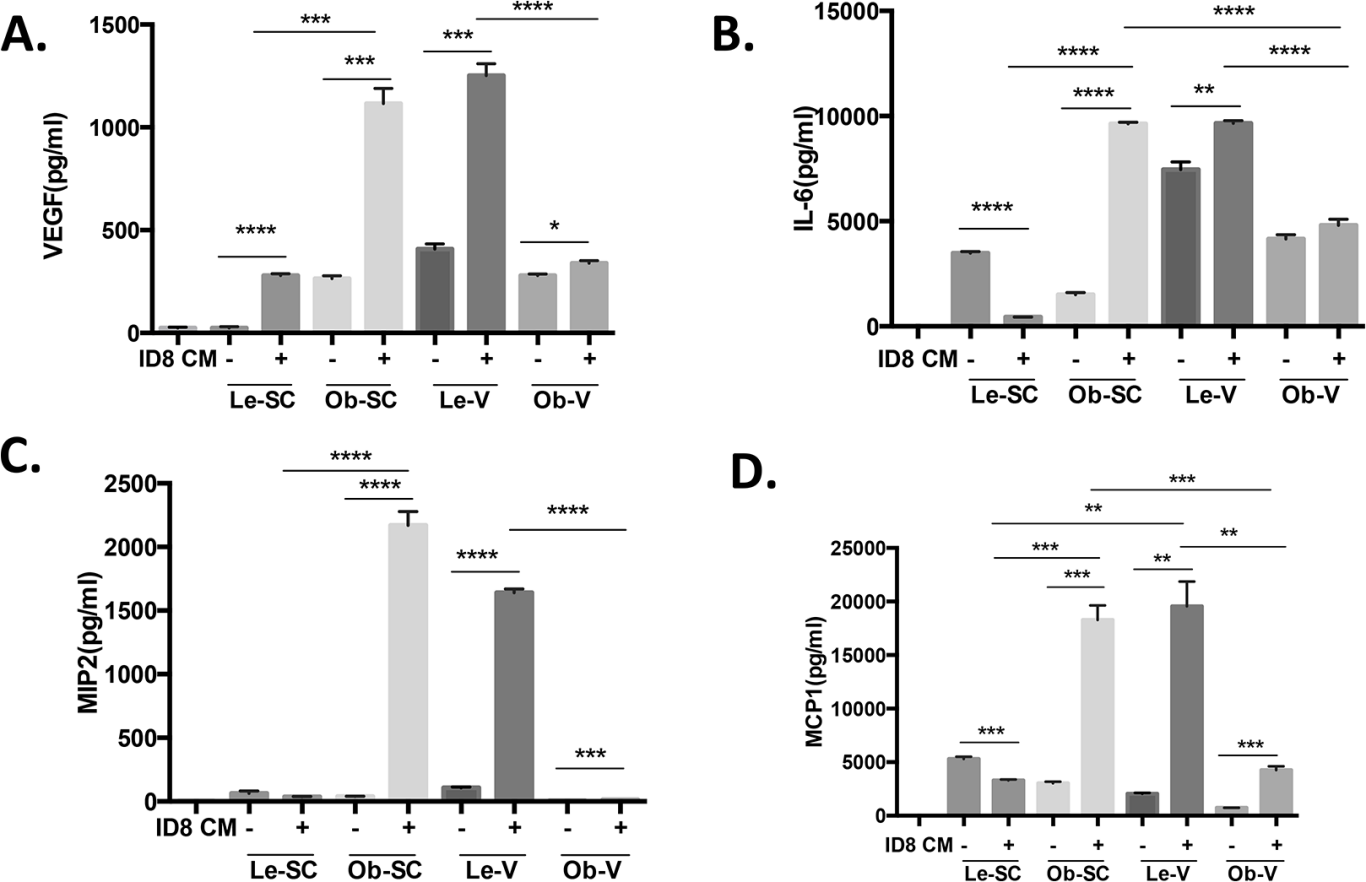
Secretion of VEGF, IL-6, MIP2 and MCP1 by ASC cells in ID8 spheroid-CM. ASC were plated at a density of 20,000/ml in cultured in spheroid medium(control) or medium contained 50% ID8 spheroid-CM for 5 days. Supernatant were collected for Luminex assay. Concentration of VEGF (**A**) IL-6 (**B**), MIP2(**C**) and MCP1 (**D**).(Shown are mean ± SEM. *, *P* < 0.05; **, *P* < 0.01, ***, P<0.001 and ****, P<0.0001. Student *t* test, two tailed.)

To determine how tissue source or tumor environment affects ASC-secreted factors, we analyzed the differences in growth factors and cytokines secreted by distinct ASC types by Luminex assay (Fig 6). ASC spheroid-CM was analyzed for angiogenic and chemotactic cytokines, including VEGF, IL-6, MCP-1, and MIP-2. Expression of these chemotactic cytokines was compared at baseline and following incubation of ASC in ID8 spheroid-CM. ID8 spheroid-CM augmented ASC secretion of VEGF from all sources (Fig 6A–6D). MIP-2, and MCP-1 secretion was up regulated by Ob-V-ASC but not Le-SC-ASC. These findings suggest that Le-SC-ASC are less responsive to tumor secreted factors which lead to up regulation of chemotactic factors by ASC from obese and visceral sources which may account for our observation that tumors from mice injected with Le-SC-ASC have fewer infiltrating inflammatory cells.

## Discussion

In this ovarian cancer model, we found that obesity dramatically promoted the growth of ovarian cancers. We provide novel evidence that this effect may be attributed, at least in part, to changes in the ASC phenotype with obesity. We found that tumors derived from mice receiving obese or visceral derived ASC grew more rapidly than controls, while lean subcutaneous derived ASC had no significant effect on tumor growth. These findings suggest that visceral adipose source and obesity change the ASC phenotype.

Despite similar cell surface marker expression and morphology among ASC, we found that tissue source and obesity changed the characteristics of ASC in many ways; including reducing the rate of ASC proliferation, limiting ASC differentiation capacity, increasing ASC survival in suspension cultures and enhancing the up regulation of chemotactic cytokines in response to tumor secreted factors. It is worth noting that the majority of studies investigating ASC have utilized ASC derived from subcutaneous adipose tissue, most commonly from lean donors. We found that Le-SC-ASC differentiated much more robustly than obese or visceral derived ASC into adipocytes and osteocytes, which may have implications for studies aimed at manipulating ASC for tissue engineering. Other authors have reported that visceral derived ASC exhibit impaired differentiation potential using clinical samples [23]. This has been proposed to be due to an intrinsic defect in IGF-I activation of the AKT pathway that may account for the impaired capacity for differentiation and proliferation of omental pre-adipocytes compared with SC-ASC [24]. We found that mice injected with Le-SC-ASC contained more intratumoral adipocytes, suggesting that increased differentiation potential seen in-vitro was seen in-vivo as well. These Le-SC-ASC–treated tumors containing more peritumoral adipocytes grew more slowly, comparable with control tumors, suggesting that ASC effects on cancer progression cannot be attributed to intra-tumoral adipocyte differentiation. Adipocyte-derived lipids have been reported to directly affect malignant cells [25]. However, we did not detect any evidence of lipid in the ID8 cells from our study.

The effect of adipose deposits on ASC phenotype is consistent with our previous studies in which we used human ASC from the omentum, rather than SC-ASC, to demonstrate that V-ASC having particularly potent effects on tumor progression, leading to enhanced tumor vascularization and promoting the survival and proliferation of tumor cells [11].

Le-SC-ASC did not up regulate expression of MCP-1, MIP-2 and IL-6, unlike obese and visceral derived ASC, and in response to tumor secreted factors. MCP-1 (CCL2) increases monocyte, T-cell and dendritic cell infiltrate while IL-6 increases myeloid derived suppressor cells [26] and MIP-2 (CXCL2) increases neutrophils recruitment [27]. These factors play a critical role in remodeling of the tumor microenvironment and are associated with cancer progression and metastasis [28] [29–31]. The lack of chemotactic cytokines secretion by Le-SC-ASC may account for the lower number of T-cells in Le-SC-ASC tumors.

Our study showed the discrepancy between the in-vitro and in-vivo effects of ASC on tumor growth, suggesting that the microenvironment plays a critical role. ASC, tumor cells and the surrounding microenvironment are closely related and interact constantly in a direct or in directly way. More macrophages detected in ASC co-injected groups may indicate that ASC could be involved in recruiting macrophages into the tumor environment. One function of IL-6 is that it can switch the differentiation of monocytes from dendritic cells to macrophages [32]. In our study, IL-6 was highly secreted in Ob-SC-ASC and Le-V-ASC than Ob-V-ASC when cultured in ID8-CM.So more macrophages in Ob-SC-ASC and Le-V-ASC co-injected groups could due to the IL-6 secretion. Walter [33] et al discovered that IL-6 secreted from adipose stromal cells promotes migration and invasion of breast cancer cells. Therefore, the role of IL-6 in ASC-macrophage interaction and its role in tumor microenvironment is worth investigating more. On the other hand, we observed Ob-SC-ASC and Le-V-ASC more MCP-1 than the other two ASC groups. Kinda et al [34] found that increase in MCP-1 expression in the adipose tissue contributes to the macrophage infiltration into the tissue, which may explain why Ob-SC-ASC and Le-V-ASC coinjected groups containing more macrophages in the tumor section. Cancer-associated macrophages (CAM) can exhibit either an M1 or M2 subtype, with M2 macrophages generally supporting cancer progression. Ovarian cancer ascites has been shown to polarize macrophages toward the M2 phenotype [35], which may promote growth and vascularization of ovarian cancers by secreting various growth factors. Furthermore, M2 macrophages in ovarian cancers have been associated with lower rates of survival [36]. Macrophages have also been proposed to increase tumor cell adhesion molecules on the peritoneal mesothelium through secretion of invasive proteases increasing tumor dissemination [37,38]. Subtype of macrophages and their interaction with ASC in the tumor microenvironment will be further addressed in our future study.

Interestingly, we found that the phenotype of Le-V-ASC appears to resemble Ob-SC-ASC in many ways, including the relative impact on ID8 tumor growth. This similarity could be interpreted to suggest that obesity, in a sense, leads to acquisition of visceral adipose qualities in the subcutaneous adipose compartment. Further studies will be needed to understand the relative effects of obesity on different adipose tissues depots and to understand the significance of these changes.

In conclusion, these results demonstrate that obese and visceral derived ASC have a unique phenotype, with limited differentiation capacity and increased expression of chemotactic factors that may account for observed effects on tumor growth.

## Supporting Information

S1 Table Statistical results of ASC surface marker expression detected by flow cytometer. ASC surface markers expression detected by flow cytometer was calculated by both percentage and mean fluorescence intensity. Experiments were repeated three times.(Shown as mean ± SEM. *, *P* < 0.05, compared with Le-SC-ASC Student *t t*est.)
(PPTX)

S1 Fig Flow cytometric characterization of ASC based of surface marker expression. Cell surface marker expression was characterized in triplicate with flow cytometry after cells were passaged 3 times. Light gray lines, isotype controls.
(PPTX)

S2 Fig Cell growth curve of SC-ASC and V-ASC from obese and lean mice. 150,000 cells were plated onto 6-well plates. Three randomly selected wells were chosen for cell numbers counting manually at day 1,4,7,11 and 14. Experiments were repeated twice. There were no significant differences in cell growth rate (Shown are mean ± SEM. P>0.05,Student *t* test, two tailed).
(PPTX)

S3 Fig Ovarian cancer cell ID8 and IG10 proliferation in co-culture with ASC and migration in response to ASC conditioned media. A, B, Proliferation of ID8/IG10 cells expressing firefly luciferase was quantified with bioluminescent imaging. There was a trend showing that growth of ID8/IG10 cancer cells in the presence of ASC increased compared with non-ASC control (gray line, P>0.05,Student *t* test, two tailed.). C, D, Migration of ID8/IG10 cells in response to ASC conditioned media. Migration assays were performed in transwell plates with 8μm pores for 8 hours with use of conditioned ASC serum-free media. Quantification analysis showed significantly more migrated cells with ASC-CM groups than with control (Shown are mean ± SEM. **, *P* < 0.01, Student *t* test, two tailed).
(PPTX)

S4 Fig Tumor cell proliferation, vascularization, adipogenesis and immune cells infiltration in ID8 tumor alone group. Nuclei are blue and vessels were stained with GSL I-isolectin B4 in red in all images (magnification is 20x). Anti-Ki67,anti-perilipin and anti-F4/80 antibodies were stained for proliferating cells, adipocytes and macrophages in green, respectively.
(PPTX)
