# Correction: Stromal Cells Derived from Visceral and Obese Adipose Tissue Promote Growth of Ovarian Cancers

**DOI:** 10.1371/journal.pone.0143019

**Published:** 2015-11-10

**Authors:** The PLOS ONE Staff

The following information is missing from the Funding section: This study was supported a grant from the Cancer Prevention and Research Institute of Texas (CPRIT, RP140609) to AHK. The publisher apologizes for this error.

## References

[1] Zhang Y, Nowicka A, Solley TN, Wei C, Parikh A, Court L, et al (2015) Stromal Cells Derived from Visceral and Obese Adipose Tissue Promote Growth of Ovarian Cancers. PLoS ONE 10(8): e0136361 doi: 10.1371/journal.pone.0136361 26317219 10.1371/journal.pone.0136361 PMC4552684

