# Supplementary material for: Stromal Cells Derived from Visceral and Obese Adipose Tissue Promote Growth of Ovarian Cancers
Source: PLoS One. 2015 Aug 28;10(8):e0136361. doi: 10.1371/journal.pone.0136361 (PMC4552684; doi:10.1371/journal.pone.0136361)
Supplement: S1 Table — ASC surface markers expression detected by flow cytometer was calculated by both percentage and mean fluorescence intensity. Experiments were repeated three times.(Shown as mean ± SEM. *, P < 0.05, compared with Le-SC-ASC Student t test.) (PPTX) [file pone.0136361.s001.pptx]

## Slide 1
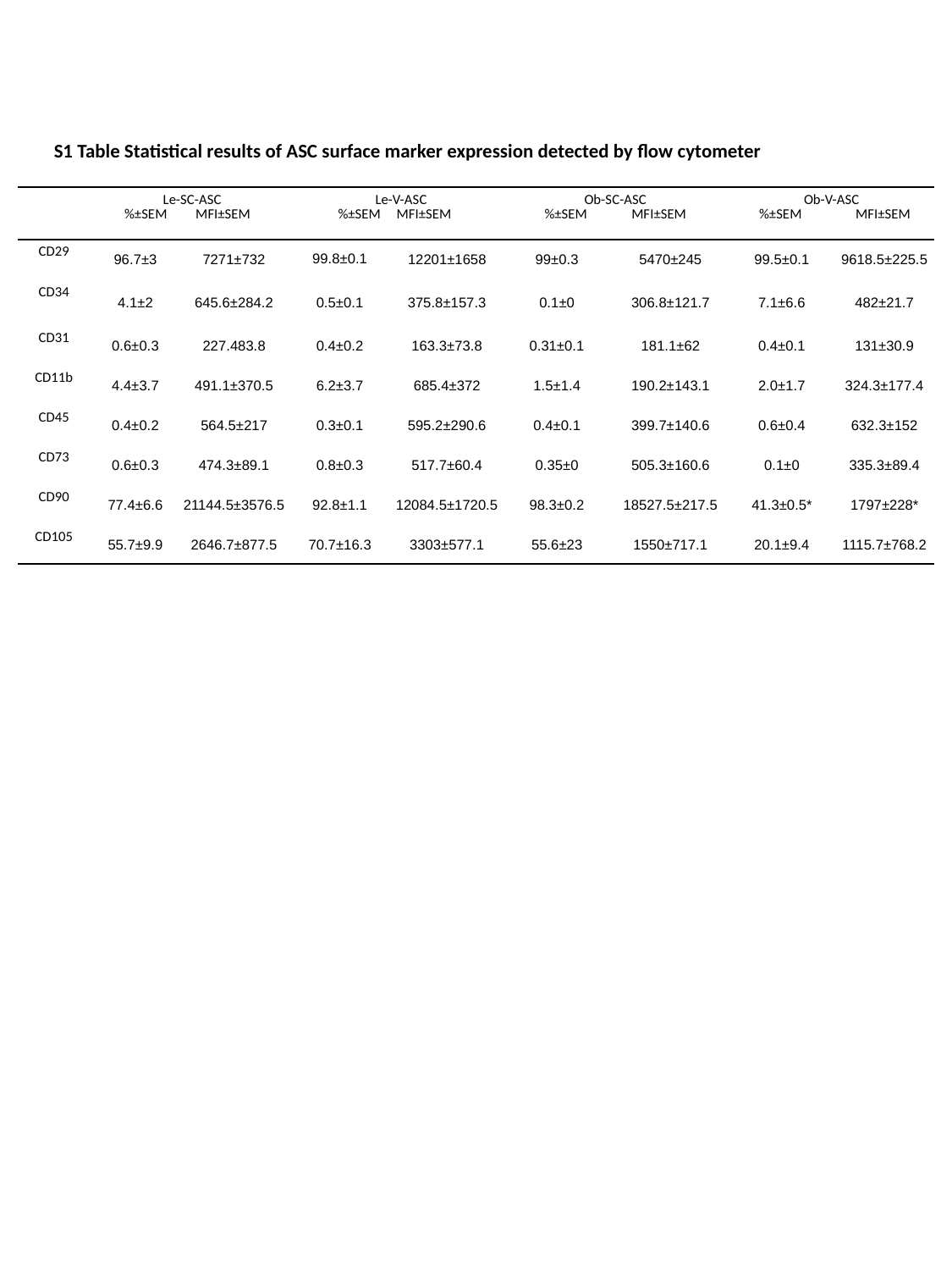

S1 Table Statistical results of ASC surface marker expression detected by flow cytometer
| | Le-SC-ASC %±SEM MFI±SEM | | Le-V-ASC %±SEM MFI±SEM | | Ob-SC-ASC %±SEM MFI±SEM | | Ob-V-ASC %±SEM MFI±SEM | |
| --- | --- | --- | --- | --- | --- | --- | --- | --- |
| CD29 | 96.7±3 | 7271±732 | 99.8±0.1 | 12201±1658 | 99±0.3 | 5470±245 | 99.5±0.1 | 9618.5±225.5 |
| CD34 | 4.1±2 | 645.6±284.2 | 0.5±0.1 | 375.8±157.3 | 0.1±0 | 306.8±121.7 | 7.1±6.6 | 482±21.7 |
| CD31 | 0.6±0.3 | 227.483.8 | 0.4±0.2 | 163.3±73.8 | 0.31±0.1 | 181.1±62 | 0.4±0.1 | 131±30.9 |
| CD11b | 4.4±3.7 | 491.1±370.5 | 6.2±3.7 | 685.4±372 | 1.5±1.4 | 190.2±143.1 | 2.0±1.7 | 324.3±177.4 |
| CD45 | 0.4±0.2 | 564.5±217 | 0.3±0.1 | 595.2±290.6 | 0.4±0.1 | 399.7±140.6 | 0.6±0.4 | 632.3±152 |
| CD73 | 0.6±0.3 | 474.3±89.1 | 0.8±0.3 | 517.7±60.4 | 0.35±0 | 505.3±160.6 | 0.1±0 | 335.3±89.4 |
| CD90 | 77.4±6.6 | 21144.5±3576.5 | 92.8±1.1 | 12084.5±1720.5 | 98.3±0.2 | 18527.5±217.5 | 41.3±0.5\* | 1797±228\* |
| CD105 | 55.7±9.9 | 2646.7±877.5 | 70.7±16.3 | 3303±577.1 | 55.6±23 | 1550±717.1 | 20.1±9.4 | 1115.7±768.2 |
