# Supplementary material for: Stromal Cells Derived from Visceral and Obese Adipose Tissue Promote Growth of Ovarian Cancers
Source: PLoS One. 2015 Aug 28;10(8):e0136361. doi: 10.1371/journal.pone.0136361 (PMC4552684; doi:10.1371/journal.pone.0136361)
Supplement: S1 Fig — Cell surface marker expression was characterized in triplicate with flow cytometry after cells were passaged 3 times. Light gray lines, isotype controls. (PPTX) [file pone.0136361.s002.pptx]

## Slide 1
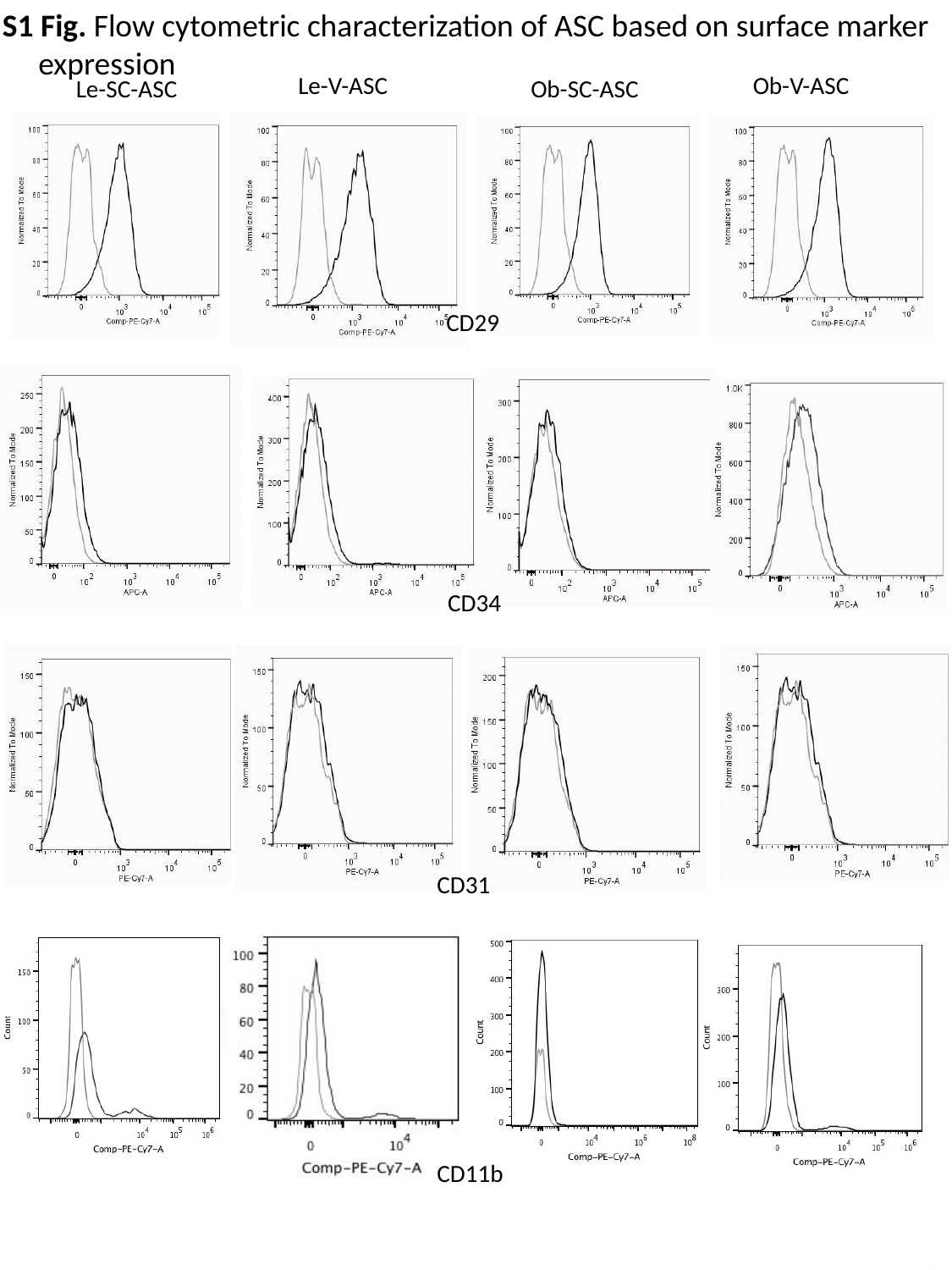

S1 Fig. Flow cytometric characterization of ASC based on surface marker expression
Ob-V-ASC
Le-V-ASC
Le-SC-ASC
Ob-SC-ASC
CD29
CD34
CD31
CD11b
1

## Slide 2
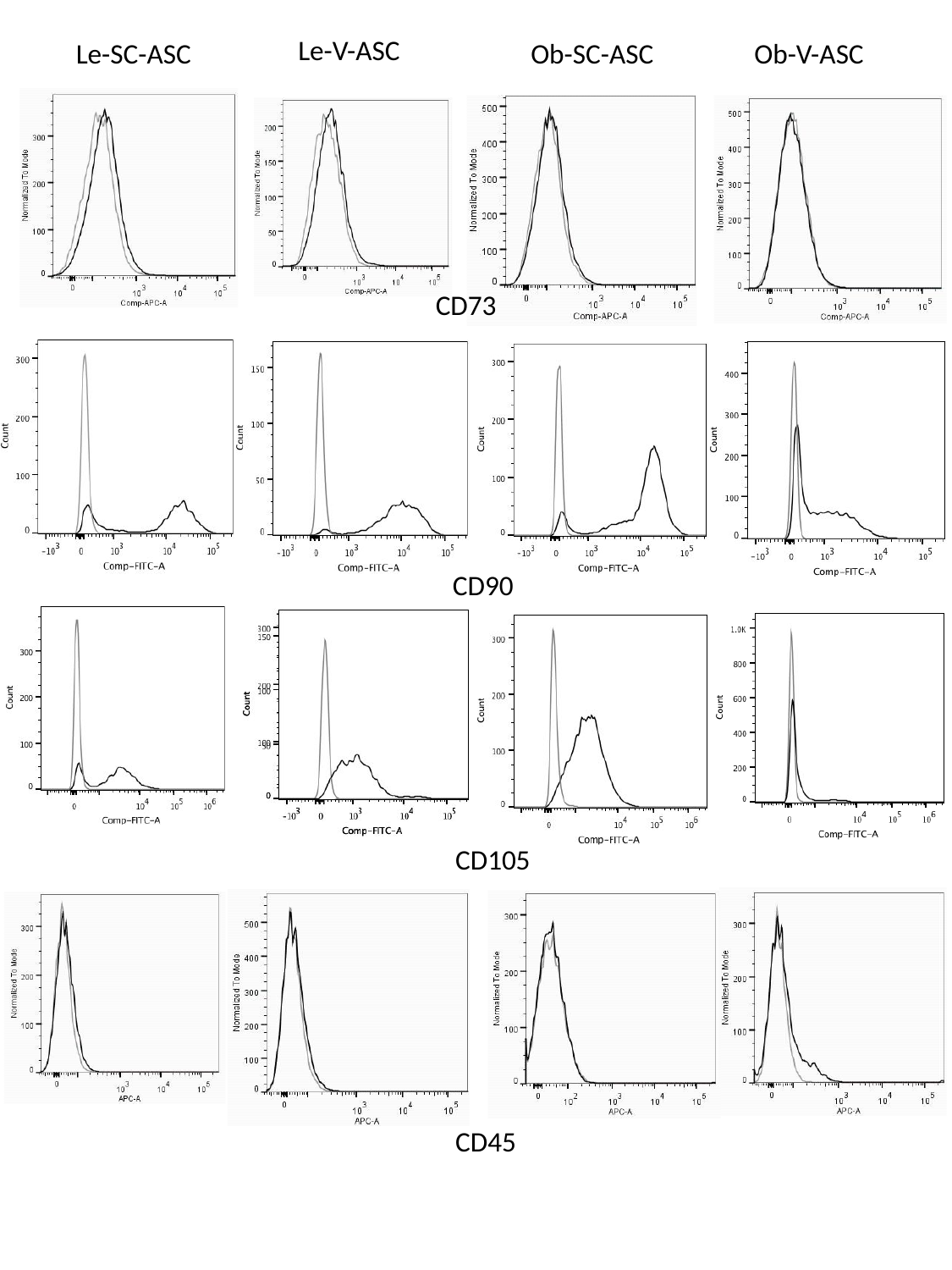

Le-V-ASC
Le-SC-ASC
Ob-SC-ASC
Ob-V-ASC
CD73
CD90
CD105
CD45
