# Supplementary figures and images for: Stromal Cells Derived from Visceral and Obese Adipose Tissue Promote Growth of Ovarian Cancers
Source: PLoS One. 2015 Aug 28;10(8):e0136361. doi: 10.1371/journal.pone.0136361 (PMC4552684; doi:10.1371/journal.pone.0136361)

## Slide 1
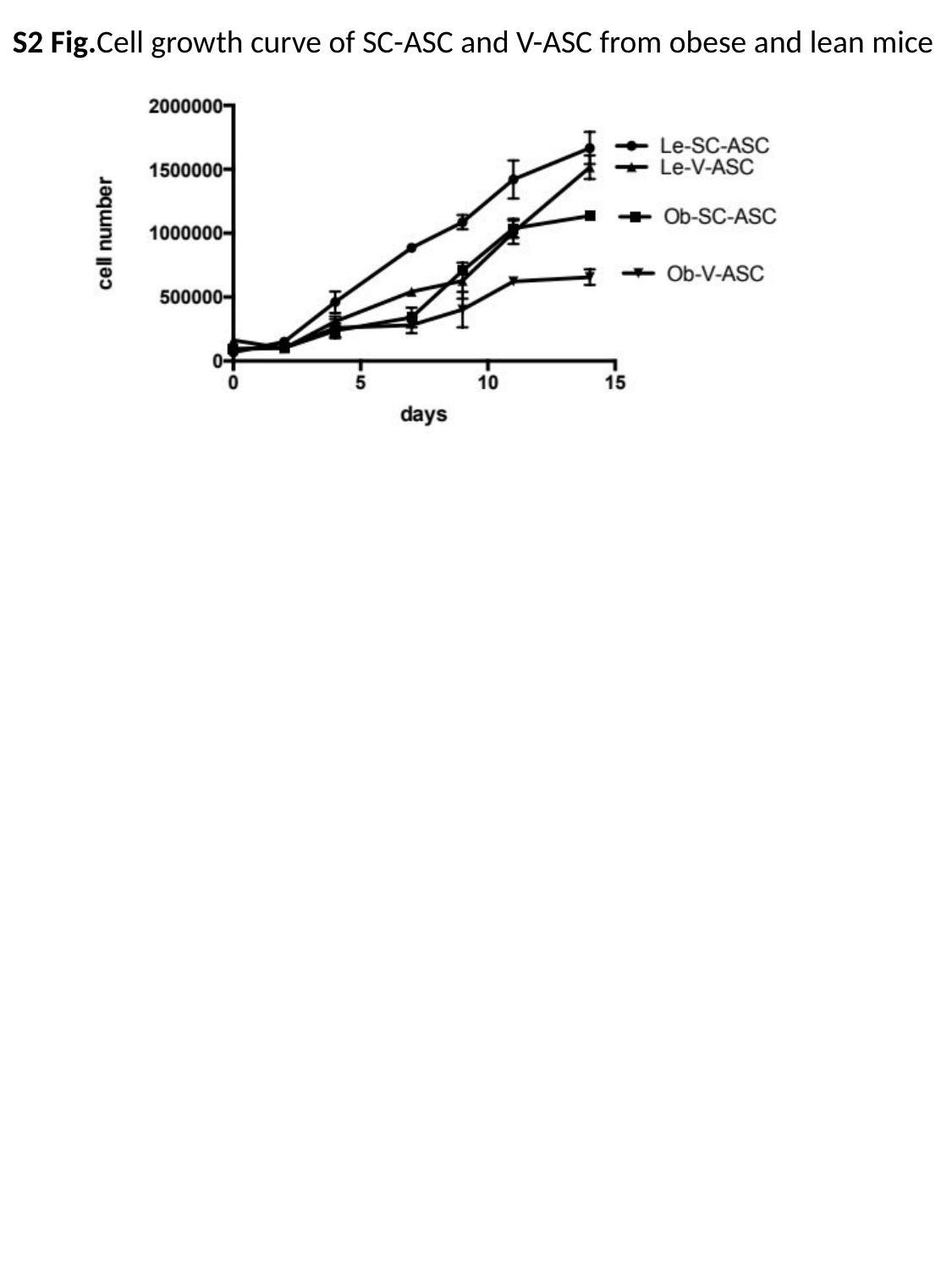

S2 Fig.Cell growth curve of SC-ASC and V-ASC from obese and lean mice

Supplement: S2 Fig — 150,000 cells were plated onto 6-well plates. Three randomly selected wells were chosen for cell numbers counting manually at day 1,4,7,11 and 14. Experiments were repeated twice. There were no significant differences in cell growth rate (Shown are mean ± SEM. P>0.05,Student t test, two tailed). (PPTX) [file pone.0136361.s003.pptx]
