# Supplementary material for: Stromal Cells Derived from Visceral and Obese Adipose Tissue Promote Growth of Ovarian Cancers
Source: PLoS One. 2015 Aug 28;10(8):e0136361. doi: 10.1371/journal.pone.0136361 (PMC4552684; doi:10.1371/journal.pone.0136361)
Supplement: S3 Fig — A, B, Proliferation of ID8/IG10 cells expressing firefly luciferase was quantified with bioluminescent imaging. There was a trend showing that growth of ID8/IG10 cancer cells in the presence of ASC increased compared with non-ASC control (gray line, P>0.05,Student t test, two tailed.). C, D, Migration of ID8/IG10 cells in response to ASC conditioned media. Migration assays were performed in transwell plates with 8μm pores for 8 hours with use of conditioned ASC serum-free media. Quantification analysis showed significantly more migrated cells with ASC-CM groups than with control (Shown are mean ± SEM. **, P < 0.01, Student t test, two tailed). (PPTX) [file pone.0136361.s004.pptx]

## Slide 1
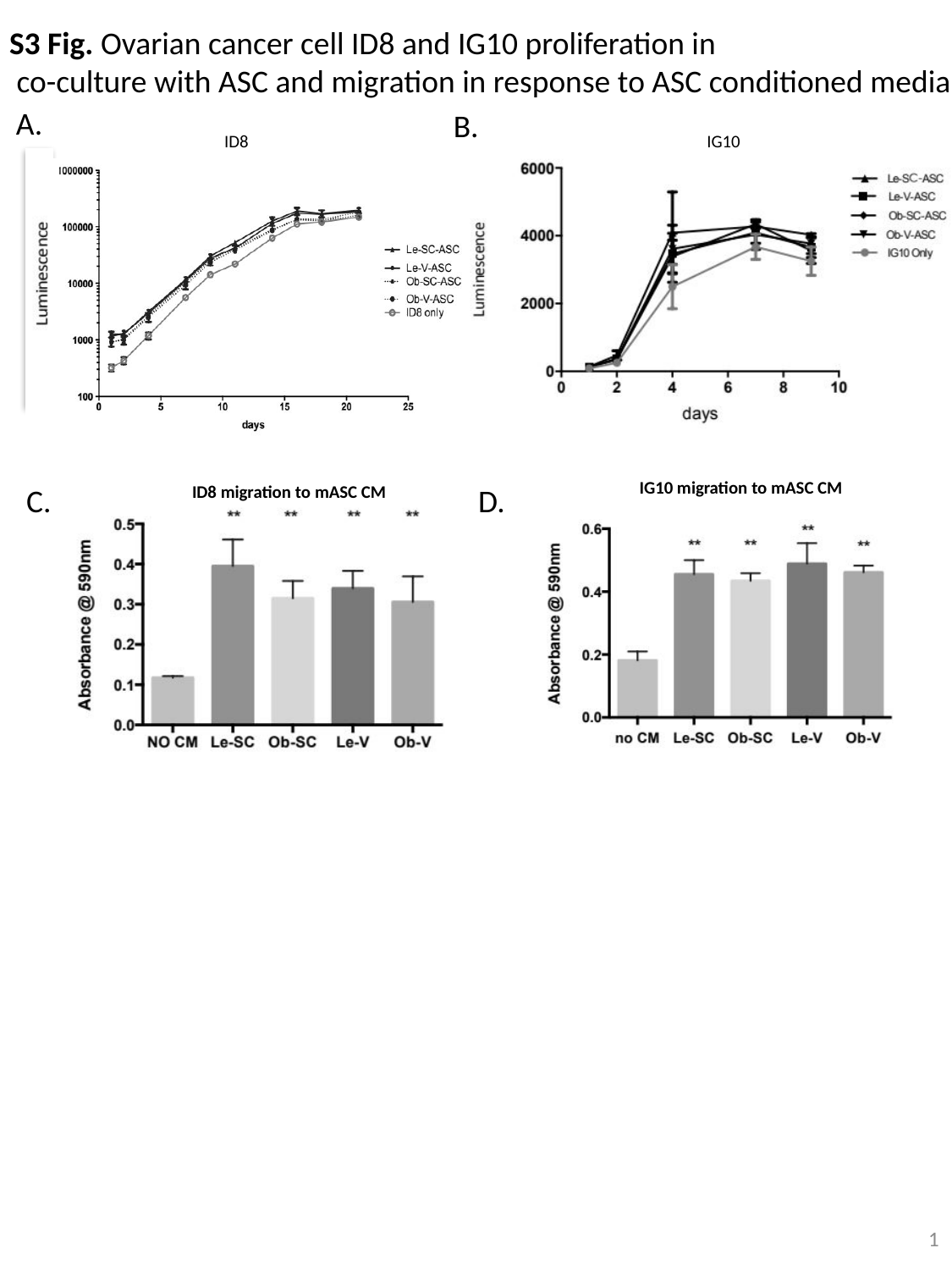

S3 Fig. Ovarian cancer cell ID8 and IG10 proliferation in
 co-culture with ASC and migration in response to ASC conditioned media
A.
B.
ID8
IG10
IG10 migration to mASC CM
C.
ID8 migration to mASC CM
D.
1
