# Supplementary material for: Stromal Cells Derived from Visceral and Obese Adipose Tissue Promote Growth of Ovarian Cancers
Source: PLoS One. 2015 Aug 28;10(8):e0136361. doi: 10.1371/journal.pone.0136361 (PMC4552684; doi:10.1371/journal.pone.0136361)
Supplement: S4 Fig — Nuclei are blue and vessels were stained with GSL I-isolectin B4 in red in all images (magnification is 20x). Anti-Ki67,anti-perilipin and anti-F4/80 antibodies were stained for proliferating cells, adipocytes and macrophages in green, respectively. (PPTX) [file pone.0136361.s005.pptx]

## Slide 1
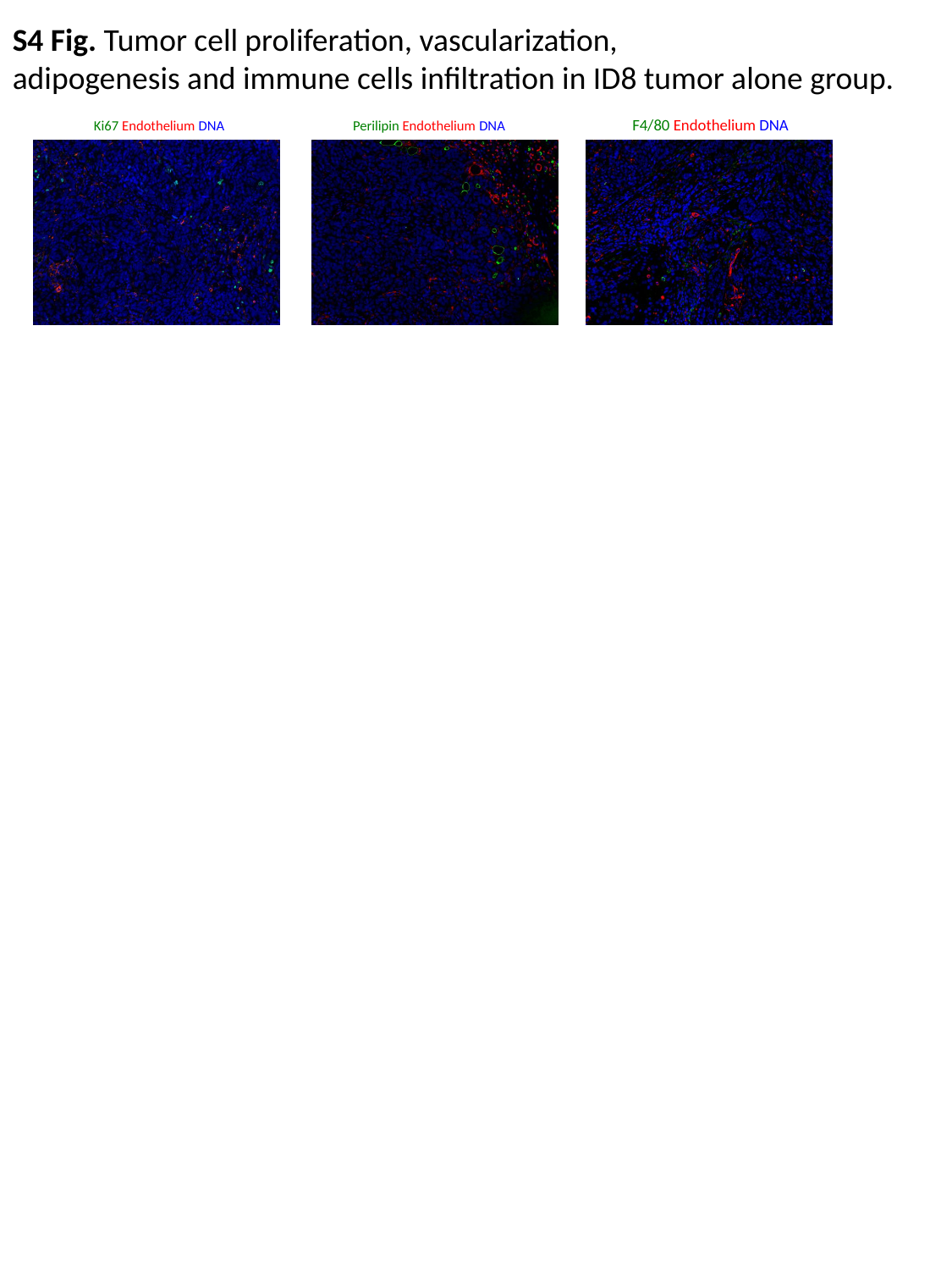

S4 Fig. Tumor cell proliferation, vascularization,
adipogenesis and immune cells infiltration in ID8 tumor alone group.
F4/80 Endothelium DNA
Ki67 Endothelium DNA
Perilipin Endothelium DNA
